# Association between fluoride exposure and the risk of serum CK and CK-MB elevation in adults: a cross-sectional study in China

**DOI:** 10.3389/fpubh.2024.1410056

**Published:** 2025-01-29

**Authors:** Junhua Wu, Ming Qin, Yue Gao, Yang Liu, Xiaona Liu, Yuting Jiang, Yanmei Yang, Yanhui Gao

**Affiliations:** Center for Endemic Disease Control, Chinese Center for Disease Control and Prevention, Harbin Medical University, Harbin, China

**Keywords:** creatine kinase, creatine kinase isoenzyme, fluoride, myocardial ischemia, arrhythmia

## Abstract

**Background:**

This study aimed to investigate the relationship between urinary fluoride concentration and myocardial disease.

**Methods:**

This is a cross-sectional study that was conducted in three villages in Wenshui County, Shanxi Province. A total of 737 villagers were included in this analysis. Urinary fluoride was detected using a fluoride-ion selective electrode. Myocardial enzymes were detected using an automatic biochemical analyzer. Myocardial ischemia and arrhythmia were diagnosed using 12-lead electrocardiogram.

**Results:**

The median level of urinary fluoride concentration was 1.32 mg/L. Urinary fluoride was associated with serum creatine kinase (CK) elevation (odds ratio [OR] = 1.39 [95% confidence interval (CI)]: 1.09–1.78) and CK isoenzyme (CK-MB) elevation (OR = 1.49 [95% CI: 1.12–1.97]). Stratified analysis revealed that urinary fluoride concentration was associated with CK elevation in villagers under the age of 60 years (OR = 1.80 [95% CI: 1.26–2.59]). This study found that there was a positive association between urinary fluoride concentration and the risk of CK-MB elevation in participants under the age of 60 years(OR = 2.18 [95% CI: 1.39–3.42]), those who were of female gender (OR = 1.53 [95% CI: 1.07–2.19]), those who were overweight/obese (OR = 1.96 [95% CI: 1.28–2.99]), those who had central obesity (OR = 1.59 [95% CI: 1.12–2.25]), consumed alcohol (OR = 1.49 [95% CI: 1.09–2.05]), and smoked (OR = 1.50 [95% CI: 1.10–2.04]).

**Conclusion:**

Our study suggests that fluoride exposure is associated with the risk of serum CK and CK-MB elevation; however, it is not associated with myocardial ischemia, arrhythmia, serum lactate dehydrogenase (LDH), serum alpha-hydroxybutyrate dehydrogenase (*α*-HBD), or serum aspartate aminotransferase (AST). Further investigations are needed to substantiate our findings and explore the potential underlying mechanisms.

## 1 Introduction

Excessive fluoride exposure is identified by the World Health Organization as one of the top ten chemicals that pose significant public health problems (1). Long-term exposure to excessive fluoride can result in fluorosis, and it may cause systemic health problems, including skeletal damage, such as dental fluorosis and skeletal fluorosis (2), and non-skeletal damage affecting the cardiovascular system, renal function, and the nervous system, among others (3). Of particular concern is cardiovascular damage that can cause a heavy disease burden in populations with high fluoride exposure, potentially becoming a public health concern in areas endemic to fluorosis (4).

An increasing number of human epidemiological studies have linked fluoride exposure to several cardiovascular diseases. Some studies have reported a positive relationship between fluoride levels in drinking water and the prevalence of hypertension, specifically high systolic blood pressure (5–7). A systematic review reports that high fluoride exposure can increase thyroid stimulating hormone (TSH) release, which may rise the risk of cardiovascular diseases (8). A cross-sectional study reveals a significant positive relationship between excessive fluoride exposure and the prevalence of carotid artery atherosclerosis (9). Previous studies have confirmed that elastic properties of the aorta are impaired in fluorosis patients and that fluorosis patients have left ventricular diastolic and global dysfunctions despite normal left ventricular systolic function (10, 11).

A study involving 61 patients establishes a positive relationship between fluoride uptake by the coronary arteries and cardiovascular risk (12). Moreover, some studies have revealed the association between fluoride exposure and myocardial disease. A case of fluoride poisoning in children is presented as ventricular arrhythmias (13). A hospital-based study has also confirmed that fluoride could develop arrhythmias in children with fluorosis (14). However, a cohort study in Sweden investigates that long-term drinking-water fluoride exposure is not associated with myocardial infarction (15). To the best of our knowledge, no study focuses on the relationship between excessive fluoride exposure and myocardial ischemia or arrhythmias in adults.

Meanwhile, some human epidemiological studies have focused on the relationship between fluoride intake from drinking water and myocardial function biomarkers. The National Health and Nutrition Examination Survey (2013–2016) among United States adolescents finds that fluoride intake from drinking water does not associate with the serum aspartate aminotransferase (AST) level (16). An epidemiological study on children reports that serum lactate dehydrogenase (LDH) activity is associated with fluoride intake from drinking water (17). However, no human epidemiological studies have investigated the relationship between fluoride intake from drinking water and the other effective indicators of cardiac function in adults, such as creatine kinase (CK), CK isoenzyme (CK-MB), and alpha-hydroxybutyrate dehydrogenase (*α*-HBD).

Therefore, this cross-sectional study, conducted in areas of Shanxi Province, China, where fluoride levels in drinking water are elevated, aims to investigate the relationship between fluoride exposure and myocardial disease, specifically in relation to myocardial enzymes in adults.

## 2 Materials and methods

### 2.1 Study population

Three villages—Gaoche, Xishe, and Xihan—located in Wenshui County, Shanxi Province, China, were selected as the investigation sites based on long-term monitoring conducted by the Shanxi Institute of Endemic Disease Prevention and Control. The fluoride concentration of drinking water in Xishe village was 1.45 mg/L. In comparison, it was 1.5 mg/L in both Gaoche and Xihan villages, exceeding the Chinese government’s stipulated limit for drinking water standards (1.2 mg/L). The inclusion criteria for our study were as follows: Villagers aged 18 years or above, who were born and have lived in these three villages. A total of 1,096 villagers were included. The exclusion criteria were as follows: (i) Villagers with diseases related to the heart, liver, muscle, or bone and had taken related medications in recent weeks (*n* = 1); (ii) villagers who did not provide a fasting blood sample (*n* = 311); (iii) villagers who did not provide their urinary sample (*n* = 47). In total, 737 villagers were enrolled for subsequent analysis.

### 2.2 General and physical information collection

General and physical information, including demographic data (age, sex, education, family income, alcohol consumption, and smoking), and disease history, were collected by trained doctoral and postgraduate students using face-to-face interviews. The height, weight, and waist circumference (WC) were also measured by trained doctoral and postgraduate students based on the Chinese government’s weight control healthcare service standards (GB/T 34821–2017). Blood pressure was measured thrice in the morning using an electronic sphygmomanometer. The body mass index (BMI) was calculated based on the height and weight.

### 2.3 Sample collection, determination, and quality control

Nurses collected 5 mL of fasting peripheral blood samples from each participant. The blood sample was centrifuged at 3,000 rpm for 10 min in 2 h, and the serum was transferred into 1.5-ml Eppendorf (EP, Corning Incorporated, New York, USA) tubes to detect myocardial function biomarkers and blood glucose levels. A 5-ml morning urine sample was also collected from each participant. All serum and urine samples were stored at −80°C in a refrigerator until analysis.

Urinary fluoride, an accepted internal measurement index of fluoride exposure (18), was detected using fluoride-ion selective electrodes according to the industry-standard method in China (WS/T 89–2015, Beijing, China). Each sample was analyzed twice, and the average result was used as the final urinary fluoride concentration.

Serum CK, serum CK-MB, serum LDH, serum *α*-HBD, serum AST, and blood glucose were measured using an automatic biochemical analyzer 3,100 (Hitachi Hi-TECH international TRADE Co., LTD, Shanghai, China). The reagent used for the measurement was provided by MedicalSystem Biotechnology Co. Ltd. (Ningbo, China), and the tests were performed according to standard operating procedures (details are shown at https://www.nbmksw.com/). Cut-off points of elevation for each myocardial enzyme are shown in Supplementary Table S1. These points are based on the industry standard of reference intervals for common clinical biochemistry tests in China (WS/T 404.1-2012, Beijing, China) and a previous study (19).

### 2.4 Diagnosis of diabetes mellitus, hypertension, skeletal fluorosis, and cardiac abnormality

Diabetes mellitus and hypertension were diagnosed through fasting blood glucose measurements and blood pressure readings. The cut-off points of fasting blood glucose for diabetes mellitus was 6.1 mmol/L, which was recommended by the Guideline for the Prevention and Treatment of Type 2 Diabetes Mellitus in China (2020 edition). The cut-off points of hypertension was 140/90 mm Hg, which was recommended by the Chinese Hypertension League Guidelines on Ambulatory Blood Pressure Monitoring (2020). Standard simultaneous 12-lead electrocardiogram (ECG) examinations were recorded at a sampling rate of 10,000 Hz (MedEx-1694, Beijing Madix Technology Co. Ltd, Beijing, China) and stored for subsequent analysis. The same technician conducted all ECG examinations and the diagnoses were made by a cardiologist and a specially trained ECG healthcare professional. Myocardial ischemia and arrhythmia were diagnosed based on the previous studies (20, 21). Skeletal fluorosis was diagnosed following the Chinese Diagnostic Criteria of Endemic Skeletal Fluorosis (WS 192–2008, Beijing, China).

### 2.5 Statistical analysis

Mean ± standard deviation (SD) or median (P25–P75) was employed to describe the continuous variables, and the categorical variables were expressed as numbers (percentages). The normal distribution test for the levels of each myocardial function biomarker was conducted by P–P chart. The urinary fluoride concentration was divided into four categorical values based on quartile and was used for further statistical analyses. Based on our prior knowledge and the directed acyclic graph, age, sex, educational level, family income, alcohol consumption, smoking, BMI, WC, diabetes mellitus, and hypertension were selected as potential confounders (Supplementary Figure S1). Binary logistic regression models were used to investigate the relationship between urinary fluoride concentration and myocardial damage.

Stratified analyses by age (<60 years and ≥ 60 years), sex (male and female), BMI (normal and overweight/obesity), WC (normal and central obesity), alcohol consumption (yes and no), and smoking (yes and no) were conducted. Furthermore, sensitivity analyses were used to test the robustness of the main results, while participants who had diabetes mellitus or hypertension were excluded.

Data analyses were performed using R statistical software (version 4.2.1; R Core Team, New Jersey, USA) and Statistical Package for the Social Sciences (SPSS) version 23.0 for Windows (SPSS, Inc., Chicago, IL, United States), and two-sided *p*-values less than 0.05 were considered statistically significant.

## 3 Results

### 3.1 Participant characteristics

The demographic statistics of 737 participants are presented in Table 1. The mean (±SD) age of the participants was 57.78 (±11.54) years and the age of 47.20% of them were above 60 years. There were more female individuals (67.17%) in this study than male counterparts (32.83%). The proportion of individuals with higher educational levels was low, those with primary or lower accounted for 36.50%, the junior high school graduates were 53.03%, and the senior high school or higher were only 10.47%. Approximately 19.75% of participants were alcohol consumers and smokers. In addition, the family income of 61.90% of participants was above 10,000 ¥/year.

**Table 1:** Basic characteristics of general population (*n* = 737).

Characteristics	All observations
Age (y, means ± SD)	57.78 ± 11.54
< 60	387 (52.80)
≥ 60	346 (47.20)
Sex (*n*, %)	
Male	241 (32.83)
Female	493 (67.17)
Educational level (*n*, %)	
Primary and below	265 (36.50)
Junior high school	385 (53.03)
Senior high school and above	76 (10.47)
Family income (*n*, %)	
< 10 000 ¥/year	277 (38.10)
≥ 10 000 ¥/year	450 (61.90)
Alcohol drinking	
No	589 (80.24)
Yes	145 (19.75)
Smoking	
No	589 (80.24)
Yes	145 (19.75)
Hypertension (*n*, %)	
Yes	282 (38.79)
No	445 (61.21)
Diabetes mellitus (*n*, %)	
Yes	61 (8.29)
No	675 (91.71)
Skeletal fluorosis (*n*, %)	
Yes	238 (32.47)
No	495 (67.53)
BMI (kg/m^2^, means ± SD)	25.70 ± 3.60
Normal	242 (32.97)
Overweight/obesity	492 (67.03)
Waistline (cm, means ± SD)	87.35 ± 9.55
Normal	198 (27.05)
Central obesity	534 (72.95)
Urinary fluoride (mg/L, median, P25-P75)	1.32 (0.90, 1.81)
Myocardial disease (*n*, %)	
Myocardial ischemia	88 (12.21)
Arrhythmia	38 (5.27)
AST (U/L, median, P25-P75)	21.50 (18.30, 26.20)
AST elevation	46 (6.35)
CK (U/L, median, P25-P75)	98.00 (71.00, 131.00)
CK elevation	66 (9.11)
CK-MB (U/L, median, P25-P75)	14.90 (12.90, 17.90)
CK-MB elevation	51 (7.02)
α-HBD (U/L, median, P25-P75)	156.50 (140.50, 174.03)
α-HBD elevation	122 (16.80)
LDH (U/L, median, P25-P75)	191.80 (174.05, 214.73)
LDH elevation	60 (8.26)

The number of AST, CK, CK-MB is 727, and α-HBD, LDH is 726.

Physical statistics found that 38.79% of participants had hypertension, 8.29% of participants had diabetes mellitus, and 32.47% of participants had skeletal fluorosis. The mean (±SD) BMI of participants was 25.70 (±3.60) kg/m^2^ and 67.03% of participants were found to be overweight and obese. In addition, the mean (±SD) WC was 87.35 (±9.55) cm, and 72.95% of participants had central obesity. ECG examinations found that 88 (12.21%) participants had myocardial ischemia and 38 (5.27%) participants had arrhythmia.

Urinary fluoride, serum levels of myocardial enzymes, and the proportion of myocardial enzyme elevation were also shown in Table 1. The median (P25–P75) level of serum CK, CK-MB, LDH, *α*-HBD, and AST were 98.00 (71.00–131.00) U/L, 14.90 (12.90–17.90) U/L, 191.80 (174.05–214.73) U/L, 156.50 (140.50–174.03) U/L, and 21.50 (18.30–26.20) U/L. The level of urinary fluoride concentration was 1.32 (0.90–1.81) mg/L.

### 3.2 Association between urinary fluoride concentrations and the risk of myocardial damage

The relationship between urinary fluoride concentration and the risk of myocardial damage is shown in Table 2. Binary logistic regression analysis found that the urinary fluoride concentration was positively associated with the risk of serum CK elevation (OR = 1.39 [95% CI: 1.09–1.78]) and CK-MB elevation (OR = 1.49 [95% CI: 1.12–1.97]).

**Table 2:** Associations between urinary fluoride concentrations and the risk of myocardial damage.

Outcomes	Urinary fluoride			*P* for trend
	Q_2_	Q_3_	Q_4_	
Myocardial ischemia	1.07 (0.52, 2.21)	0.95 (0.46, 1.97)	1.53 (0.77, 3.03)	0.242
Arrhythmia	0.90 (0.35, 2.33)	0.63 (0.23, 1.72)	0.72 (0.27, 1.94)	0.404
CK	1.81 (0.79, 4.17).	1.68 (0.72, 3.90)	**3.09 (1.39, 6.87)**	**0.008**
CK-MB	1.72 (0.65, 4.55)	1.33 (0.48, 3.70)	**3.66 (1.47, 9.09)**	**0.006**
LDH	0.36 (0.13, 0.99)	1.06 (0.49, 2.30)	1.16 (0.55, 2.47)	0.245
α-HBD	1.01 (0.54, 1.90)	1.30 (0.71, 2.38)	1.22 (0.67, 2.24)	0.384
AST	0.61 (0.23, 1.65)	1.17 (0.50, 2.75)	1.11 (0.46, 2.68)	0.523

Models were adjusted for age, sex, educational level, family income, BMI, waistline, alcohol drinking, smoking, hypertension and diabetes mellitus. Q_1_ as reference group. The bold values means the difference among groups have statistical significant.

Sensitivity analysis showed that the urinary fluoride concentration was also positively associated with the risk of serum CK elevation (OR = 1.73 [95%CI, 1.25–2.39]) and CK-MB elevation (OR = 1.80 [95% CI: 1.23–2.63]) when excluding participants with hypertension (Supplementary Table S2). In addition, when excluding participants with diabetes mellitus, we still found a positive association between urinary fluoride concentration and the risk of serum CK elevation (OR = 1.56 [95% CI: 1.20–2.02]) and CK-MB elevation (OR = 1.55 [95% CI: 1.15–2.10]) (Supplementary Table S3).

### 3.3 Relationship between urinary fluoride concentration and the risk of CK and CK-MB elevation in different subgroups

Stratified analysis was conducted in subgroups according to age, sex, BMI, WC, alcohol consumption, and smoking, with the goal of revealing the association between urinary fluoride concentration and the risk of CK elevation, as presented in Table 3. There was a positive association between urinary fluoride concentration and the risk of serum CK elevation in participants under the age of 60 years (OR = 1.80 [95% CI: 1.26–2.59]). In addition, no interaction effect was found between urinary fluoride concentration and the remaining subgroups regarding the risk of serum CK elevation.

**Table 3:** Associations between urinary fluoride concentrations and the risk of CK elevation in subgroups.

Subgroup	No of events (%)	Urinary fluoride			*P-*interaction
		Q_2_	Q_3_	Q_4_	
Age (years)					0.29
< 60	35 (9.19)	**7.21 (1.51, 34.40)**	**5.57 (1.15, 26.96)**	**12.79 (2.69, 60.93)**	
≥ 60	31 (9.06)	0.64 (0.19, 2.10)	0.74 (0.23, 2.31)	1.22 (0.45, 3.23)	
Sex					0.75
Male	26 (11.02)	2.37 (0.64, 8.75)	2.05 (0.52, 8.09)	2.88 (0.75, 11.13)	
Female	40 (8.20)	1.55 (0.52, 4.69)	1.52 (0.52, 4.48)	**2.95 (1.09, 8.00)**	
BMI (kg/m^2^)					0.25
Normal	21 (8.90)	4.39 (0.83, 23.17)	3.36 (0.56, 20.14)	**7.94 (1.42, 44.45)**	
Overweight/obesity	45 (9.28)	1.36 (0.49, 3.78)	1.42 (0.53, 3.80)	2.39 (0.94, 6.10)	
Waistline (cm)					0.11
Normal	14 (7.14)	**10.90 (1.13, 104.95)**	3.32 (0.26, 42.07)	**17.69 (1.59, 197.27)**	
Central obesity	52 (9.89)	1.31 (0.50, 3.41)	1.64 (0.66, 4.08)	**2.53 (1.06, 6.04)**	
Alcohol drinking					0.75
No	52 (8.93)	1.39 (0.53, 3.62)	1.61 (0.64, 4.06)	**2.97 (1.22, 7.19)**	
Yes	14 (9.86)	4.44 (0.69, 28.57)	2.09 (0.24, 18.19)	4.21 (0.59, 30.07)	
Smoking					0.98
No	54 (9.26)	1.54 (0.61, 3.91)	1.83 (0.75, 4.44)	**2.75 (1.17, 6.48)**	
Yes	12 (8.51)	6.14 (0.59, 63.95)	1.01 (0.05, 19.72)	**15.79 (1.23, 203.35)**	

Models were adjusted for age, sex, educational level, family income, BMI, waistline, alcohol drinking, smoking, hypertension and diabetes mellitus, with stratified variables not adjusted in the stratified analysis. *P-*interaction values was adjusted by FDR - corrected. Q_1_ as reference group. The bold values means the difference among groups have statistical significant.

The same stratified analysis was conducted to assess the risk of CK-MB elevation in Table 4. There was a positive association between urinary fluoride concentration and the risk of CK-MB elevation in participants under the age of 60 years (OR = 2.18 [95% CI: 1.39–3.42]), female (OR = 1.53 [95% CI: 1.07–2.19]), overweight/obesity (OR = 1.96 [95% CI: 1.28–2.99]), central obesity (OR = 1.59 [95% CI: 1.12–2.25]), alcohol consumption (OR = 1.49 [95% CI: 1.09–2.05]), and smoking (OR = 1.50 [95% CI: 1.10–2.04]). No interaction effects were observed on the risk of serum CK-MB elevation.

**Table 4:** Associations between urinary fluoride concentrations and the risk of CK-MB elevation in subgroups.

Subgroup	No of events (%)	Urinary fluoride			*P-*interaction
		Q_2_	Q_3_	Q_4_	
Age (years)					0.26
< 60	26 (6.82)	1.37 (0.29, 6.59)	2.89 (0.71, 11.85)	**8.70 (2.19, 34.54)**	
≥ 60	25 (7.31)	1.71 (0.48, 6.17)	0.39 (0.07, 2.27)	1.92 (0.56, 6.56)	
Sex					0.99
Male	18 (7.63)	2.83 (0.53, 15.25)	2.16 (0.36, 12.90)	4.12 (0.74, 22.84)	
Female	33 (6.76)	1.30 (0.38, 4.48)	0.98 (0.27, 3.60)	**3.46 (1.17, 10.22)**	
BMI (kg/m^2^)					0.99
Normal	24 (10.08)	1.69 (0.47, 6.10)	1.68 (0.42, 6.77)	1.68 (0.42, 6.73)	
Overweight/obesity	27 (5.56)	1.55 (0.33, 7.24)	1.29 (0.28, 6.06)	**6.22 (1.69, 22.97)**	
Waistline (cm)					0.99
Normal	17 (8.67)	3.15 (0.51, 19.37)	4.38 (0.70, 27.45)	3.39 (0.51, 22.45)	
Central obesity	34 (6.46)	1.41 (0.42, 4.68)	0.83 (0.21, 3.22)	**3.84 (1.32, 11.20)**	
Alcohol drinking					0.99
No	42 (6.34)	1.41 (0.48, 4.16)	1.29 (0.43, 3.88)	**3.39 (1.25, 9.17)**	
Yes	9 (7.22)	3.84 (0.31, 48.14)	1.21 (0.06, 25.06)	8.84 (0.70, 110.96)	
Smoking					0.93
No	42 (6.38)	1.29 (0.45, 3.73)	0.95 (0.31, 2.92)	**3.35 (1.32, 8.50)**	
Yes	9 (7.20)	-	-	-	

Models were adjusted for age, sex, educational level, family income, BMI, waistline, alcohol drinking, smoking, hypertension and diabetes mellitus, with stratified variables not adjusted in the stratified analysis. *P-*interaction values was adjusted by FDR - corrected. Q_1_ as reference group. The bold values means the difference among groups have statistical significant.

## 4 Discussion

Cell experiments revealed that fluoride could induce damage in H9c2 cardiomyocytes (22), and myocardial damage would lead to a change in the level of myocardial enzymes. Some studies have reported that an association between fluoride exposure and myocardial infarction, serum CK, and LDH, although their conclusions remain inconsistent. In addition to CK and LDH, the most common myocardial enzyme spectrum in clinical practice include CK-MB, *α*-HBD, and AST (23, 24). Therefore, our study focused not only on the relationship between urinary fluoride concentration and the risk of myocardial enzymes (serum CK, serum CK-MB, serum LDH, serum α-HBD, and serum AST) but also on myocardial ischemia and arrhythmia in a population-based cross-sectional study. We found that urinary fluoride concentration was only positively associated with the risk of serum CK and CK-MB elevation; however, it was not associated with myocardial ischemia, arrhythmia, or the remaining myocardial enzymes.

Our research found the urinary fluoride concentration was not associated with myocardial ischemia, which was consistent with the study conducted in a large cohort study of 455,619 people in Sweden (15). However, our findings regarding the relationship between urinary fluoride concentration and arrhythmia were inconsistent with previous studies conducted on children with acute fluoride poisoning or chronic fluorosis (13, 14). These inconsistent findings of arrhythmia might be caused by the different doses of fluoride intake, duration of exposure, and specific age group.

There were three tissue- and compartment-specific CK isoenzymes, including CK-BB (brain), CK-MM (skeletal muscle), and CK-MB (cardiac muscle), which mainly catalyzed the reversible conversion of creatine and ATP to phosphocreatine and adenosine diphosphate (25). Previous studies have reported considerable controversy regarding the relationship between fluoride and CK. Animal experiments revealed that fluoride did not affect CK’s activity *in vitro* (26). However, it also found that the activity of serum CK was significantly increased with sodium fluoride in another animal experiment (27). Our study’s binary logistic regression analysis indicated that the urinary fluoride concentration was positively associated with serum CK elevation. Moreover, at the same time, this kind of positive relationship was confirmed by the sensitivity analyses in participants without diabetes or hypertension. According to these analyses, it prompted that the fluoride could be associated with estimated myocardial damage. In addition, our study found that the urinary fluoride concentration was positively associated with serum CK elevation in people under the age of 60 years. It might be caused by more physical labor and muscle in people under the age of 60 years. Hence, further research should be conducted to determine the potential underlying mechanisms of this effect.

As a type of CK isoenzyme, CK-MB had higher sensitivity in detecting acute myocardial infarction, which was close to 100% (28). Animal experiments revealed that sodium fluoride intervention (set at 300 mg/mL for 10 days) could increase serum CK-MB in male rats (29). Coincidentally, this relationship was also confirmed in a study involving a group of male rats exposed to high doses of sodium fluoride (administered at 45 and 90 mg F-/kg body weight/24 hours treated rats) (30). To the best of our knowledge, there were no studies focused on the relationship between urinary fluoride concentration and serum CK-MB elevation in a natural population. In addition, in contrast to previous studies that focused on male animals, our study found that fluoride exposure was a risk factor for CK-MB elevation in females rather than males. It might be caused by the different concentrations of calcium between males and females, and further studies on female rats should be conducted. Moreover, we also found that fluoride exposure was a risk factor for CK-MB elevation in participants without alcohol consumption or smoking, which the sex distribution might cause. Previous studies revealed that obesity was a risk factor for cardiomyopathy caused by the calcium homeostasis disequilibrium in mitochondria and oxidative stress (31). Our research also found that fluoride exposure was a risk factor for serum CK-MB elevation in obese participants, which the additive effect of fluoride exposure and obesity might cause.

Epidemiological studies in children found that serum LDH activity was associated with drinking water fluoride in children (17), and this relationship was also found in cell and animal experiments (9, 26, 32, 33). However, our study found no significant association between urinary fluoride concentration and the risk of serum LDH elevation. This inconsistent result in the population study might be due to the potential confounders we had controlled in our research, but not in those previous studies. *α*-HBD comprised the total activity of some LDH isoenzymes, namely LDH1 and LDH2, which were mainly found in myocardial damage (34, 35). As a serological biomarker for myocardial alteration, no studies that investigated the relationship between fluoride and *α*-HBD before. Hence, our study first reported that fluoride exposure in the population could not increase the risk of serum α-HBD elevation.

AST was still the most important biomarker for myocardial injury (36). Our study found there was no association between urinary fluoride concentration and the risk of AST elevation, which was consistent with a study based on 1,742 adolescents (16).

Our research had some limitations. First, our study was a natural population cross-sectional investigation, which could not determine the exact causality between urinary fluoride concentration and the risk of serum CK, CK-MB elevation. Second, some known cardiovascular-related confounders were not included in our research. Meanwhile, the participants in our research might consume fluoride in different ways than drinking water, such as food, which needs further evaluation. Third, our assessment of fluoride exposure was limited to urinary fluoride measurements, thereby excluding other potential sources of exposure, such as drinking water and dietary intake. Finally, this was a single-center study and did not include the different types of fluorosis, such as brick-tea-type fluorosis or chronic coal-burning fluorosis.

## 5 Conclusion

In conclusion, our study provides population-based evidence for the relationship between urinary fluoride concentration and myocardial disease related to myocardial enzymes, including CK, CK-MB, LDH, *α*-HBD, and AST. Notably, fluoride exposure may be associated with the risk of serum CK and CK-MB elevation in adults but not with myocardial ischemia, arrhythmia, serum LDH, serum α-HBD, and serum AST. Further investigations are needed to substantiate our findings, elucidate the potential mechanism underlying fluoride-induced elevation of CK and CK-MB, and explore how fluoride-induced changes in myocardial enzymes may affect myocardial injury.

## Data Availability

The original contributions presented in the study are included in the article/Supplementary material, further inquiries can be directed to the corresponding authors.

## References

[1] World Health Organization. Lead poisoning and health. (2018) Available at: http://www.who.int/news-room/fact-sheets/detail/lead-poisoning-and-health (Accessed March 05, 2020).

[2] Veneri F Iamandii I Vinceti M Birnbaum LS Generali L Consolo U. Fluoride exposure and skeletal fluorosis: a systematic review and dose-response Meta-analysis. Curr Environ Health Rep. (2023) 10:417–41. doi: 10.1007/s40572-023-00412-9, PMID: 37861949

[3] Zhou J Sun D Wei W. Necessity to pay attention to the effects of low fluoride on human health: an overview of skeletal and non-skeletal damages in epidemiologic investigations and laboratory studies. Biol Trace Elem Res. (2023) 201:1627–38. doi: 10.1007/s12011-022-03302-7, PMID: 35661326

[4] Kurdi MS. Chronic fluorosis: the disease and its anaesthetic implications. Indian J Anaesth. (2016) 60:157–62. doi: 10.4103/0019-5049.177867, PMID: 27053777 PMC4800930

[5] Koh S Park S. The association between fluoride in water and blood pressure in children and adolescents. Pediatr Res. (2022) 92:1767–72. doi: 10.1038/s41390-022-01982-4, PMID: 35190682

[6] Sun L Gao Y Liu H Zhang W Ding Y Li B. An assessment of the relationship between excess fluoride intake from drinking water and essential hypertension in adults residing in fluoride endemic areas. Sci Total Environ. (2013) 443:864–9. doi: 10.1016/j.scitotenv.2012.11.021, PMID: 23246666

[7] Liu Y Téllez-Rojo M Sánchez BN Ettinger AS Osorio-Yáñez C Solano M. Association between fluoride exposure and cardiometabolic risk in peripubertal Mexican children. Environ Int. (2020) 134:105302. doi: 10.1016/j.envint.2019.105302, PMID: 31726363 PMC6904509

[8] Iamandii I De Pasquale L Giannone ME Veneri F Generali L Consolo U. Does fluoride exposure affect thyroid function? A systematic review and dose-response meta-analysis. Environ Res. (2024) 242:117759. doi: 10.1016/j.envres.2023.117759, PMID: 38029816

[9] Liu H Zeng Q Cui Y Yu L Zhao L Hou C. The effects and underlying mechanism of excessive iodide on excessive fluoride-induced thyroid cytotoxicity. Environ Toxicol Pharmacol. (2014) 38:332–40. doi: 10.1016/j.etap.2014.06.008, PMID: 25104093

[10] Varol E Akcay S Ersoy IH Koroglu BK Varol S. Impact of chronic fluorosis on left ventricular diastolic and global functions. Sci Total Environ. (2010) 408:2295–8. doi: 10.1016/j.scitotenv.2010.02.011, PMID: 20206377

[11] Varol E Akcay S Ersoy IH Ozaydin M Koroglu BK Varol S. Aortic elasticity is impaired in patients with endemic fluorosis. Biol Trace Elem Res. (2010) 133:121–7. doi: 10.1007/s12011-009-8578-4, PMID: 20012382

[12] Li Y Berenji GR Shaba WF Tafti B Yevdayev E Dadparvar S. Association of vascular fluoride uptake with vascular calcification and coronary artery disease. Nucl Med Commun. (2012) 33:14–20. doi: 10.1097/MNM.0b013e32834c187e, PMID: 21946616

[13] Yolken R Konecny P McCarthy P. Acute fluoride poisoning. Pediatrics. (1976) 58:90–3. doi: 10.1542/peds.58.1.90 934788

[14] Karademir S Akçam M Kuybulu AE Olgar S Oktem F. Effects of fluorosis on QT dispersion, heart rate variability and echocardiographic parameters in children. Anadolu Kardiyol Derg. (2011) 11:150–5. doi: 10.5152/akd.2011.038, PMID: 21342861

[15] Näsman P Granath F Ekstrand J Ekbom A Sandborgh-Englund G Fored CM. Natural fluoride in drinking water and myocardial infarction: a cohort study in Sweden. Sci Total Environ. (2016) 562:305–11. doi: 10.1016/j.scitotenv.2016.03.161, PMID: 27100011

[16] Malin AJ Lesseur C Busgang SA Curtin P Wright RO Sanders AP. Fluoride exposure and kidney and liver function among adolescents in the United States: NHANES, 2013-2016. Environ Int. (2019) 132:105012. doi: 10.1016/j.envint.2019.105012, PMID: 31402058 PMC6754771

[17] Xiong X Liu J He W Xia T He P Chen XM. Dose-effect relationship between drinking water fluoride levels and damage to liver and kidney functions in children. Environ Res. (2007) 103:112–6. doi: 10.1016/j.envres.2006.05.008, PMID: 16834990

[18] Zhao L Yu C Lv J Cui Y Wang Y Hou C. Fluoride exposure, dopamine relative gene polymorphism and intelligence: a cross-sectional study in China. Ecotoxicol Environ Saf. (2021) 209:111826. doi: 10.1016/j.ecoenv.2020.111826, PMID: 33360592

[19] Huang S Liu Z Ge X Luo X Zhou Y Li D. Occupational exposure to manganese and risk of creatine kinase and creatine kinase-MB elevation among ferromanganese refinery workers. Am J Ind Med. (2020) 63:394–401. doi: 10.1002/ajim.23097, PMID: 32112445

[20] Elhaj FA Salim N Harris AR Swee TT Ahmed T. Arrhythmia recognition and classification using combined linear and nonlinear features of ECG signals. Comput Methods Prog Biomed. (2016) 127:52–63. doi: 10.1016/j.cmpb.2015.12.024, PMID: 27000289

[21] Ibanez B James S Agewall S Antunes MJ Bucciarelli-Ducci C Bueno H. 2017 ESC guidelines for the management of acute myocardial infarction in patients presenting with ST-segment elevation: the task force for the management of acute myocardial infarction in patients presenting with ST-segment elevation of the European Society of Cardiology (ESC). Eur Heart J. (2018) 39:119–77. doi: 10.1093/eurheartj/ehx393, PMID: 28886621

[22] Yan X Wang L Yang X Qiu Y Tian X Lv Y. Fluoride induces apoptosis in H9c2 cardiomyocytes via the mitochondrial pathway. Chemosphere. (2017) 182:159–65. doi: 10.1016/j.chemosphere.2017.05.002, PMID: 28494360

[23] Li SW Sun X He Y Guo Y Zhao HJ Hou ZJ. Assessment of arsenic trioxide in the heart of *Gallus gallus*: alterations of oxidative damage parameters, inflammatory cytokines, and cardiac enzymes. Environ Sci Pollut Res Int. (2017) 24:5781–90. doi: 10.1007/s11356-016-8223-7, PMID: 28054265

[24] Wang L Zheng M Tang Y Yin Y Liu Y Liu G. Impact of various periods of perfusion-pause and reperfusion on the severity of myocardial injury in the langenodorff model. Perfusion. (2023) 38:1609–16. doi: 10.1177/02676591221122349, PMID: 36059244

[25] Del Franco A Ambrosio G Baroncelli L Pizzorusso T Barison A Olivotto I. Creatine deficiency and heart failure. Heart Fail Rev. (2022) 27:1605–16. doi: 10.1007/s10741-021-10173-y, PMID: 34618287 PMC9388465

[26] Nedeljković M Matović V Soldatović D. Levels of lactate dehydrogenase and creatine kinase in plasma of fluoride-treated rabbits. J Appl Toxicol. (1985) 5:11–3. doi: 10.1002/jat.2550050103, PMID: 3989216

[27] Hassan HA Yousef MI. Mitigating effects of antioxidant properties of black berry juice on sodium fluoride induced hepatotoxicity and oxidative stress in rats. Food Chem Toxicol. (2009) 47:2332–7. doi: 10.1016/j.fct.2009.06.023, PMID: 19540898

[28] Mair J Morandell D Genser N Lechleitner P Dienstl F Puschendorf B. Equivalent early sensitivities of myoglobin, creatine kinase MB mass, creatine kinase isoform ratios, and cardiac troponins I and T for acute myocardial infarction. Clin Chem. (1995) 41:1266–72. doi: 10.1093/clinchem/41.9.1266, PMID: 7656437

[29] Oyagbemi AA Omobowale TO Asenuga ER Adejumobi AO Ajibade TO Ige TM. Sodium fluoride induces hypertension and cardiac complications through generation of reactive oxygen species and activation of nuclear factor kappa beta. Environ Toxicol. (2017) 32:1089–101. doi: 10.1002/tox.22306, PMID: 27378751

[30] Panneerselvam L Govindarajan V Ameeramja J Nair HR Perumal E. Single oral acute fluoride exposure causes changes in cardiac expression of oxidant and antioxidant enzymes, apoptotic and necrotic markers in male rats. Biochimie. (2015) 119:27–35. doi: 10.1016/j.biochi.2015.10.002, PMID: 26455266

[31] Ren J Wu NN Wang S Sowers JR Zhang Y. Obesity cardiomyopathy: evidence, mechanisms, and therapeutic implications. Physiol Rev. (2021) 101:1745–807. doi: 10.1152/physrev.00030.2020, PMID: 33949876 PMC8422427

[32] Chen L Ning H Yin Z Song X Feng Y Qin H. The effects of fluoride on neuronal function occurs via cytoskeleton damage and decreased signal transmission. Chemosphere. (2017) 185:589–94. doi: 10.1016/j.chemosphere.2017.06.128, PMID: 28719878

[33] Pal P Mukhopadhyay PK. Fluoride induced testicular toxicities in adult Wistar rats. Toxicol Mech Methods. (2021) 31:383–92. doi: 10.1080/15376516.2021.1891489, PMID: 33641618

[34] Lee S Koppensteiner R Kopp CW Gremmel T. α-Hydroxybutyrate dehydrogenase is associated with atherothrombotic events following infrainguinal angioplasty and stenting. Sci Rep. (2019) 9:18200. doi: 10.1038/s41598-019-54899-0, PMID: 31796860 PMC6890648

[35] Ruzich RS. Cardiac enzymes. How to use serial determinations to confirm acute myocardial infarction. Postgrad Med. (1992) 92:85–92. doi: 10.1080/00325481.1992.11701533, PMID: 1437917

[36] Lofthus DM Stevens SR Armstrong PW Granger CB Mahaffey KW. Pattern of liver enzyme elevations in acute ST-elevation myocardial infarction. Coron Artery Dis. (2012) 23:22–30. doi: 10.1097/MCA.0b013e32834e4ef1, PMID: 22113063

