# Supplementary material for: Association between fluoride exposure and the risk of serum CK and CK-MB elevation in adults: a cross-sectional study in China
Source: Front Public Health. 2025 Jan 29;12:1410056. doi: 10.3389/fpubh.2024.1410056 (PMC11818751; doi:10.3389/fpubh.2024.1410056)

TABLE S1. The levels of elevation for each myocardial enzymes

Myocardial enzymes		Levels of elevation
CK (U/L)		
	Female	≥ 170
	Male	≥ 194
CK-MB (U/L)		≥ 24
LDH (U/L)		≥ 245
α-HBD (U/L)		≥ 182
AST (U/L)		
	Female	≥ 35
	Male	≥ 40

TABLE S2. Sensitivity analysis estimating association between urinary fluoride concentrations and the risk of myocardial enzyme elevation, excluded hypertension

Outcomes	Urinary fluoride			*P* for trend
	Q_2	Q_3	Q_4	
Myocardial ischemia	1.31 (0.48, 3.52)	0.76 (0.26, 2.22)	1.70 (0.65, 4.48)	0.459
Arhythmia	1.35 (0.43, 4.30)	0.75 (0.22, 2.61)	0.58 (0.15, 2.33)	0.312
CK	**3.58 (1.08, 11.87)**	**3.37 (1.01, 11.32)**	**7.34 (2.27, 23.72)**	**0.001**
CK-MB	2.84 (0.70, 11.46)	2.99 (0.74, 12.07)	**7.34 (1.95, 27.62)**	**0.002**
LDH	0.34 (0.06, 2.01)	1.77 (0.53, 5.92)	1.98 (0.58, 6.77)	0.090
α-HBD	1.16 (0.46, 2.92)	1.70 (0.71, 4.04)	1.43 (0.57, 3.57)	0.310
AST	0.66 (0.22, 2.04)	0.87 (0.30, 2.50)	0.78 (0.25, 2.42)	0.752

Note: Models were adjusted for age, sex, educational level, family income, BMI, waistline, alcohol drinking, smoking and diabetes mellitus. Q_1 as reference group. The bold values means the difference among groups have statistical significant.

TABLE S3. Sensitivity analysis estimating association between urinary fluoride concentrations and the risk of myocardial enzyme elevation, excluded diabetes mellitus

Outcomes	Urinary fluoride			*P* for trend
	Q_2	Q_3	Q_4	
Myocardial ischemia	0.84 (0.38, 1.85)	0.99 (0.47, 2.09)	1.45 (0.71, 2.97)	0.220
Arhythmia	1.19 (0.43, 3.26)	0.81 (0.28, 2.33)	0.86 (0.29, 2.49)	0.605
CK	2.49 (0.97, 6.38)	2.47 (0.97, 6.32)	**4.72 (1.92, 11.59)**	**0.001**
CK-MB	1.65 (0.58, 4.72)	1.53 (0.53, 4.47)	**3.97 (1.51, 10.48)**	**0.004**
LDH	0.41 (0.15, 1.15)	1.01 (0.44, 2.33)	1.35 (0.61, 2.98)	0.161
α-HBD	0.91 (0.47, 1.77)	1.23 (0.65, 2.32)	1.18 (0.62, 2.23)	0.431
AST	0.49 (0.17, 1.41)	0.86 (0.35, 2.13)	1.03 (0.42, 2.55)	0.716

Note: Models were adjusted for age, sex, educational level, family income, BMI, waistline, alcohol drinking, smoking and hypertension. Q_1 as reference group. The bold values means the difference among groups have statistical significant.

FIGURE S1. Directed acyclic graph for the associations between urinary fluoride concentration and myocardial damage

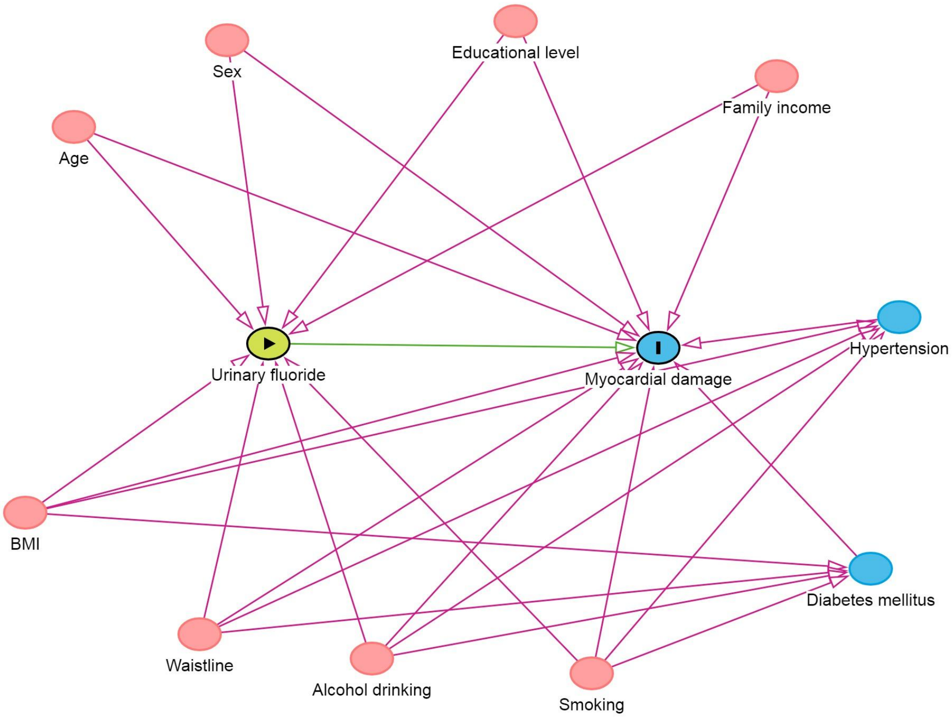

Supplement: Supplementary file 1 [file Data_Sheet_1.pdf]
